# Therapeutic and Immunologic Effects of Short-Chain Fatty Acids in Inflammatory Bowel Disease: A Systematic Review

**DOI:** 10.3390/ijms252010879

**Published:** 2024-10-10

**Authors:** Ignacio Ventura, Miryam Chomon-García, Francisco Tomás-Aguirre, Alma Palau-Ferré, María Ester Legidos-García, María Teresa Murillo-Llorente, Marcelino Pérez-Bermejo

**Affiliations:** 1 Molecular and Mitochondrial Medicine Research Group, School of Medicine and Health Sciences, Catholic University of Valencia San Vicente Mártir, C/Quevedo No. 2, 46001 Valencia, Spain; ignacio.ventura@ucv.es; 2 Translational Research Center San Alberto Magno CITSAM, Catholic University of Valencia San Vicente Mártir, C/Quevedo No. 2, 46001 Valencia, Spain; 3 School of Medicine and Health Sciences, Catholic University of Valencia San Vicente Mártir, C/Quevedo No. 2, 46001 Valencia, Spain; miryam.chomon@mail.ucv.es (M.C.-G.); paco.tomas@ucv.es (F.T.-A.); 4 SONEV Research Group, Faculty of Medicine and Health Sciences, Catholic University of Valencia San Vicente Mártir, C/Quevedo No. 2, 46001 Valencia, Spain; am.palau@ucv.es (A.P.-F.); ester.legidos@ucv.es (M.E.L.-G.); mt.murillo@ucv.es (M.T.M.-L.)

**Keywords:** inflammatory bowel disease, short-chain fatty acids, butyric acid, butyrate

## Abstract

Inflammatory bowel disease is a chronic condition characterized by recurrent intestinal inflammation. Its etiopathogenesis is driven by a series of events that disrupt the mucosal barrier, alter the healthy balance of intestinal microbiota, and abnormally stimulate intestinal immune responses. Therefore, numerous studies suggest the use of short-chain fatty acids and their immunomodulatory effects as a therapeutic approach in this disease. The objective of this systematic review was to synthesize previous evidence on the relevance and therapeutic use of short-chain fatty acids, particularly butyrate, in the immune regulation of inflammatory bowel disease. This systematic review of articles linking inflammatory bowel disease with short-chain fatty acids was conducted according to the PRISMA-2020 guidelines. The Medline and the Web of Science databases were searched in August 2024. The risk of bias was assessed using the Joanna Briggs Institute checklists. A total of 1460 articles were reviewed, of which, 29 met the inclusion criteria. Short-chain fatty acids, particularly butyrate, play a critical role in the regulation of intestinal inflammation and can be used as a strategy to increase the levels of short-chain fatty acid-producing bacteria for use in therapeutic approaches.

## 1. Introduction

Inflammatory bowel disease (IBD) is a chronic disease with recurrent intestinal inflammation. It is a general term for a group of diseases that mainly includes Crohn’s disease (CD), ulcerative colitis (UC), and unclassified inflammatory bowel disease (UC-IBD). Of unknown etiopathogenesis, it involves the inappropriate and persistent activation of the intestinal mucosal immune system. Environmental factors play a critical role in the development of inflammatory bowel disease (IBD). In particular, the adoption of a Western diet high in saturated fat and low in fiber disrupts the balance of the gut microbiota. In addition, the prolonged use of antibiotics negatively affects microbial diversity, while high levels of hygiene are associated with reduced exposure to beneficial microorganisms. These factors contribute to the development of dysbiosis, creating a pro-inflammatory environment that promotes inappropriate immune activation in genetically predisposed individuals [1].

CD can affect any part of the gastrointestinal tract, including the mouth, esophagus, stomach, small intestine, rectum, and anus. It is mainly associated with abdominal pain and problems such as fistulas, rectal lesions, and rectal bleeding, which are more common in UC and occur in areas of the large intestine, including the colon and rectum [1]. The etiopathogenesis of both diseases is caused by a series of events that disrupt the mucosal barrier, alter the healthy balance of the intestinal microbiota, and inappropriately stimulate the intestinal immune response.

It is estimated that more than 1 million people in the United States and 2.5 million people in Europe have IBD, resulting in significant health care costs [2]. The disease can begin in childhood, although the incidence usually peaks in adulthood [3]. According to several studies [4,5,6], UC is slightly more common in men (60%). However, CD is slightly more common in women, suggesting some involvement of hormonal factors in the expression of the disease. The prevalence of IBD is increasing, particularly in developed and industrialized countries. It is estimated that it will affect up to 30 million people worldwide by 2025 [7].

The gut microbiome has attracted increasing interest as a factor regulating the balance in the gut of healthy individuals. Several environmental and lifestyle factors, such as hygiene, the use of antibiotics, and the adoption of a typical Western diet, have been linked to imbalances in the gut microbiota, known as dysbiosis. This alteration of the microbiome can lead to an imbalance in the production of pro-inflammatory cytokines, such as tumor necrosis factor-alpha (TNF-α) and interleukin-6 (IL-6), and a decrease in anti-inflammatory cytokines, such as interleukin-10 (IL-10). This shift creates a chronic pro-inflammatory environment in the GI tract, disrupting intestinal homeostasis and contributing to the development of inflammatory diseases such as IBD. [8]. The gut microbiome of a healthy individual consists of a balanced community of diverse microorganisms, including bacteria, bacteriophages, viruses, archaea, and fungi. Among the bacterial species of particular importance are those that feed on indigestible dietary fiber and produce metabolites such as short-chain fatty acids (SCFAs), especially acetate, propionate, and butyrate. Butyrate acts as a major energy source for colonic cells and also contributes to the maintenance of intestinal homeostasis through its anti-inflammatory activity [8,9].

**Figure 1:**
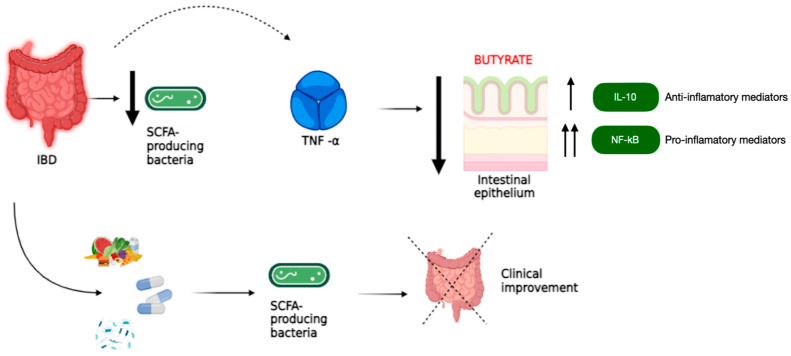
Synergistic interactions between SCFA-producing bacteria and anti-inflammatory drugs in the treatment of IBD: The figure shows the interaction between SCFA-producing bacteria and anti-inflammatory drugs in the treatment of IBD. SCFA-producing bacteria are beneficial inhabitants of the gut that ferment dietary carbohydrates to produce SCFAs such as acetate, propionate, and butyrate. These SCFAs are essential for gut health because they nourish the intestinal cells: they are the main source of energy for the cells that line the intestine, maintaining their protective function. They fight inflammation by reducing the production of inflammatory substances such as TNF-α and increasing the production of other anti-inflammatory substances such as IL-10. They also help strengthen the intestinal barrier, which protects the body from pathogens and harmful substances. The anti-inflammatory drugs that are commonly used to treat inflammation in IBD work by reducing the production of pro-inflammatory mediators, including TNF-α. SCFA-producing bacteria and anti-inflammatory drugs may have a synergistic effect in reducing inflammation and strengthening the intestinal barrier, helping to improve IBD symptoms and promote remission.

Figure 1 shows a synergistic interaction between SCFA-producing bacteria and anti-inflammatory drugs in the treatment of IBD. Anti-inflammatory drugs reduce the production of inflammatory mediators, while SCFA-producing bacteria have anti-inflammatory effects, strengthen the intestinal barrier, and promote the production of anti-inflammatory mediators. This joint interaction may improve disease symptoms and promote remission. IL-10 and NF-κB are two key molecules that regulate the inflammatory response in the gut. IL-10 acts as an inhibitor of intestinal inflammation, while NF-κB enhances it. The imbalance between these two molecules contributes to the development and progression of IBD. Therapeutic strategies that increase IL-10 or decrease NF-κB activity may be promising for the treatment of IBD. In conclusion, SCFA-producing bacteria, anti-inflammatory drugs, IL-10, and NF-κB play important roles in gut health. Synergistic interactions between SCFA-producing bacteria and anti-inflammatory drugs may be a valuable treatment option for people with IBD, while strategies that modulate IL-10 and NF-κB may also have therapeutic potential [8,9].

Butyrate is particularly important in the therapeutic use of SCFAs due to its central role in regulating intestinal inflammation and maintaining microbiome homeostasis. As the primary energy source for colonic epithelial cells, butyrate is essential for maintaining the integrity of the intestinal barrier. In addition, butyrate exerts significant anti-inflammatory effects by inhibiting histone deacetylases (HDACs), leading to epigenetic modifications in immune cells that help regulate the immune response within the gastrointestinal tract. These properties have been shown to be particularly beneficial in conditions such as IBD, where butyrate can help reduce inflammation and restore a balanced gut microbiome, making it a key SCFA in therapeutic interventions. Therefore, the aim of this study was to synthesize previous evidence on the relevance and the therapeutic use of short-chain fatty acids, especially butyrate, in the immune regulation of inflammatory bowel disease.

## 2. Materials and Methods

A systematic review was performed according to the Preferred Reporting Items for Systematic Reviews and Meta-Analyses (PRISMA) 2020 guidelines [10], which was registered in the records of the Open Science Framework (https://osf.io/9738p; accessed on 1 September 2024). The PRISMA 2020 checklist (Supplementary Data S1) was also applied.

### 2.1. Research Question and Eligibility Criteria

The review was designed to answer the following question: “What is the effect of restoring butyrate-producing bacteria by controlled administration of prebiotics, probiotics, and specific dietary treatments on the anti-inflammatory activity of short-chain fatty acids in patients with inflammatory bowel disease (IBD)?”. The PICO strategy was used, with P (population), I (intervention/exposure), C (comparison), and O (outcome) defined as follows.

#### 2.1.1. Inclusion Criteria

- Population: adults diagnosed with ulcerative colitis or Crohn’s disease.
- Intervention: the administration of prebiotics, probiotics, or dietary treatments aimed at restoring butyrate-producing bacteria.
- Comparator: comparison with patients who did not receive such interventions.
- Outcome: the evidence of anti-inflammatory effects associated with short-chain fatty acids.
- Publication language: English or Spanish.
- Publication date: articles published between January 2000 and August 2024.
- Study design: prospective or retrospective observational studies (cross-sectional, case-control, or cohort studies), reviews, case studies and case series.

#### 2.1.2. Exclusion Criteria

We excluded the following works:

- Articles that did not directly address the impact of SCFAs or their immunomodulatory role in inflammatory bowel disease.
- Letters to the editor, personal opinions, books, book chapters, non-original reports, conference abstracts, editorials, commentaries, or articles that did not contribute to or complement the objectives of the study.
- Studies involving patients with additional serious medical conditions, known allergies to prebiotics or probiotics, pregnancy, or participation in other clinical trials that could confound the results.
- Articles for which the full text could not be obtained.

### 2.2. Sources of Information and Search Strategy

A comprehensive search of the Medline and the Web of Science databases was performed in August 2024. The search strategy used the following Medical Subject Headings (MeSHs) terms and keywords: “inflammatory bowel disease”, “short-chain fatty acids”, “butyric acid”, and “butyrate”. These terms were combined using the Boolean operators “AND” and “OR”. Search strategies are reported in Supplementary Table S1.

In addition, a hand search of the grey literature and the bibliographic references of the included studies was carried out to include papers that might have been overlooked.

### 2.3. Selection of Studies

After eliminating duplicate articles, the initial screening of titles and abstracts was carried out, followed by a review of the full text. The initial screening was performed by two authors, I.V. and M.C.-G, and the full-text review was performed by all of the authors.

### 2.4. Data Extraction

The following data were extracted from the articles that were finally included for the full review: the year of publication, the country of study, sample characteristics, and measures of anti-inflammatory activity related to short-chain fatty acids.

### 2.5. Quality Assessment

Joanna Briggs Institute (JBI) checklists [11], appropriate for each type of study, were used to assess the study’s design and quality. These checklists have 4 response options: yes, no, unclear, or not applicable. Items were rated by the number of positive answers to the total number of possible answers as percentages, dividing the number of possible answers by the number of “not applicable” items. A score of less than 50% was considered to be of low quality. The quality assessment of each study was reviewed independently by two authors: I.V. and M.C.-G. Any disagreements were resolved by discussion with the other reviewers.

## 3. Results

**Figure 2:**
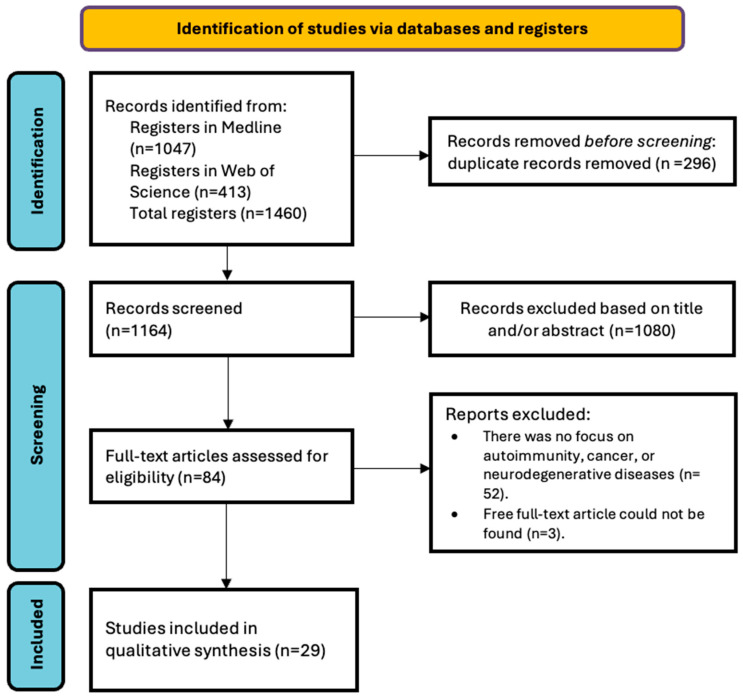
PRISMA flowchart of the study selection process.

The literature search yielded 1460 results, as shown in Figure 2. Eighty-four articles were selected for the full-text review, of which, 29 studies met the inclusion criteria, after duplicates were eliminated and the remaining articles were screened. Table 1 summarizes the results of the search of the selected studies. Supplementary Tables S2–S6 show the quality assessment of the studies.

The reviewed papers were mainly focused on evaluating the impact of different treatments and dietary supplements in IBD patients, especially in CD [12,16,19,21,22,23,27,35] and UC [24,30,31,32,33,36]. The objectives included the evaluation of the effect of prebiotics such as galactooligosaccharides (GOS) [13,14,18], pectin, and different SCFAs, such as butyrate, on gut microbiota, inflammation, and clinical parameters [14]. In addition, the effects of dietary interventions, such as the Mediterranean diet, and the efficacy of fecal microbiota transplantation (FMT) were investigated [13,16,20].

Regarding prebiotics and SCFAs, it was observed that GOS supplementation increased the presence of bifidobacteria, although it did not significantly reduce clinical scores or inflammation in UC patients [12]. On the other hand, the use of pectin together with FMT helped to maintain the composition and diversity of the intestinal microbiota [13]. Butyrate studies showed an increase in SCFA-producing bacteria and, in some cases, improvements in inflammation and clinical parameters. Diets such as CD-TREAT and the Mediterranean diet have shown beneficial changes in the microbiota and reductions in inflammation in IBD patients [17]. A low-fat, high-fiber diet increased acetate levels, benefiting the intestinal barrier and reducing inflammation. FMT studies revealed a complex relationship between microbial colonization and butyrate production [16]. Although an improvement in microbial diversity was observed, increasing the genetic capacity for butyrate production through FMT proved challenging [8].

The studies conclude that prebiotics and dietary supplements, such as butyrate, may have beneficial effects on the microbiota and reduce inflammation in IBD patients [13,14,15,18,20,25,26,28,29,32,37,38,39,40]. However, their efficacy is variable, and many studies highlight the need for additional controlled clinical trials to validate these findings. Dietary interventions show significant potential to improve symptoms and the quality of life in IBD patients, suggesting a comprehensive approach, combining specific diets with microbial therapies. In conclusion, research shows a growing interest in the role of microbiota and SCFAs in the management of IBD. Despite variations in results, there is an emerging consensus on the potential of special diets and prebiotic and butyrate supplementation to improve gut health in these patients. Most agree that it is essential to continue with larger, controlled studies to establish definitive clinical guidelines.

## 4. Discussion

The aim of this work was to investigate how the controlled manipulation of the gut microbiota, as well as the use of prebiotics, probiotics, and specific dietary treatments, can effectively modulate inflammation and, thus, improve clinical outcomes in patients with IBD. To this end, we focused on the hypothesis that the restoration of butyrate-producing bacteria appears to play a pivotal role in promoting long-term remission in UC and CD [15,19].

Studies conducted by several authors on IBD patients have shown that SCFAs, and, thus, SCFA-producing bacteria, play a beneficial role in disease control [20,21]. SCFAs have been identified as playing a critical role in this mechanism by targeting mammalian G-protein coupled receptors (GPRs), particularly GPR41 and GPR43 [37]. Accumulating evidence suggests that SCFAs may enhance T-cell regulatory (Treg) function through GPR activation and histone deacetylase (HDAC) inhibition due to epigenetic effects [37]. In summary, GPRs may protect against intestinal inflammation by maintaining the integrity of the epithelial barrier and regulating the immune response [37]. Furthermore, by analyzing the dynamics of microbial colonization and persistence in IBD patients, focusing on butyrate production gene transfer, it was concluded that butyrate production plays metabolic, regulatory, and immune functions [37]. This allows us to link the decrease in microbiota populations associated with butyrate production to IBD [16,23,26,28].

Both pectin and inulin-type fructans (ITF) are soluble fibers with prebiotic properties that are beneficial for gut health [36]. Pectin, which is fermented by the gut microbiota into SCFAs, particularly butyrate, has significant effects in the gut. Its use in UC patients undergoing fecal microbiota transplantation (FMT) not only preserves the diversity of the intestinal flora, but also enhances the effect of the transplantation [13,16,23,24,36]. On the other hand, inulin-type fructans, such as short-chain fructooligosaccharides (scFOS), are considered prebiotics and produce short-chain fatty acids when fermented in the gastrointestinal tract. The consumption of 15 g/day of inulin-type fructans during a 9-week study demonstrated significant improvements in UC markers and led to remarkable changes in the gut microbiota compared to the baseline. These results suggest that both pectin and inulin-type fructans may play an important role in the treatment and management of inflammatory bowel disease (IBD) [13,27].

Diet also emerges as an important component in the management of IBD. Three proposals have been made. The first is to study how an individualized diet based on foods rich in short-chain fatty acids can affect IBD patients [17]. This work concludes that this type of diet mimics beneficial changes in the microbiome, reduces intestinal inflammation, and is, therefore, a well-tolerated and effective diet for patients with CD. The Mediterranean diet has a higher proportion of short-chain fatty acids than other diets, such as the usual Canadian diet (CHD) [28]. This Mediterranean diet (MDP) is associated with changes in the microbiome and an improvement in intestinal inflammation and may, therefore, lead to clinical improvement in inactive UC [19]. Finally, a low-fat, high-fiber diet has been shown to reduce the markers of inflammation and dysbiosis in UC patients and improve their quality of life [24,25]. This type of diet leads to an increase in Bacteroides, which contain the main producers of acetate, thus increasing the levels of this short-chain fatty acid and having a beneficial effect on the intestinal barrier and inflammatory responses [22]. In other words, fiber intake is associated with a reduction in flares in patients with Crohn’s disease [36].

The gut microbiota plays an important role in the pathogenesis of IBD, and studying it allows us to identify the key markers of dysbiosis in these patients. Some of the signs found are decreased acetate CoA transferase, decreased absolute levels of total SCFAs, and the ratio of certain to major SCFAs [29,30,31,34]. All of these may indicate an inhibition of the functional activity and the number of anaerobic microflora and/or an alteration in the utilization of SCFAs by colonocytes, which could be used as potential targets for the development of personalized treatments for IBD patients [28,36]. One example is the use of butyrate enemas, which can prevent atrophy and inflammation in the bypassed GI mucosa of IBD patients [18]. We also found the use of sodium butyrate microcapsules as a possible potential strategy in IBD patients. The term “microcapsule” refers to a spherical body that encapsulates the active ingredient [41]. These sodium butyrate microcapsules increase the growth of SCFA-producing bacteria, resulting in benefits for these IBD patients, particularly in UC [14].

A preliminary evaluation of the butyrate and short-chain fatty acid profile was performed in patients with ulcerative colitis during a flare, who were treated with mesalamine or a combination of myrrh, chamomile flowers, and coffee charcoal. The results showed a significant reduction in the total amount of short-chain fatty acids and butyrate during a flare in the patients treated with mesalamine, whereas no significant changes were observed in the patients treated with the herbal preparation [21].

Studies by Hamilton et al. [20] show the importance of SCFA-producing bacteria in IBD. Fecal microbiota transplantation has identified the type of bacteria associated with sustained remission of UC. This procedure restores butyrate-producing bacteria and increases ButCoA levels, suggesting an important role for butyrate in long-term remission of UC. Studies of the fecal microbiota of Chinese and Japanese patients have shown that IBD is associated with a decrease in SCFA-producing bacteria [23,24]. One of the most important bacteria in this regard is *Fecalibacterium prausnitzii*. This bacterium is an acetate consumer that produces butyrate and bioactive anti-inflammatory molecules, such as shikimic acid and salicylic acid. It has been shown that high levels of *F. prausnitzii* in our microbiota lead to an improvement in the gut ecosystem and that patients with low levels of it have worse inflammatory parameters [25]. It has also been found that IBD patients have lower levels of this bacterium, resulting in reduced anti-inflammatory activities of these butyrate-producing bacteria [26,30,31,32,33,34,35,36,39,40].

Short-chain fatty acids, especially butyrate, play a key role as an energy source for the intestinal epithelium [42]. In addition, people with ulcerative colitis have significantly lower levels of total short-chain fatty acids, as well as acetate, propionate and valerate, than people without the disease [38]. This allows us to consider the possibility of using this type of bacteria as a possible treatment strategy [20]. It is important to note that not all scientific evidence supports the idea of the benefits of SCFAs, but some authors have conducted research suggesting that short-chain fatty acids have no clear benefit in IBD patients. These studies conclude that supplementation with prebiotic galactooligosaccharides (GOS) did not improve bowel motility or inflammation, although it did normalize stool [12,15]. In addition, the ratio of *Bifidobacterium* to *Christensenellaceae* was found to be increased only in patients with a less active disease, suggesting that the prebiotic effect may depend on disease status, although a controlled trial is needed to confirm these observations [12]. The trial of sodium butyrate supplementation for 12 weeks as an adjunctive therapy in newly diagnosed children and adolescents also failed to show efficacy in IBD [15].

Finally, studies of the epithelial response to butyrate have shown that the response to this SCFA is not intrinsically altered in resting IBD patients, supporting the beneficial effects of butyrate in the absence of inflammation. Nevertheless, due to the downregulation of butyrate transporters and enzymes associated with both active UC and CD patients, supplementation with butyrate extracts or SCFAs may not have beneficial effects as long as inflammation persists, highlighting the need for further studies in this area [29,31].

The studies reviewed have shown the relationship between SCFA-producing bacteria, anti-inflammatory drugs, IL-10, and NF-κB on gut health. SCFA-producing bacteria are beneficial bacteria in the gut that ferment dietary carbohydrates to produce SCFAs such as acetate, propionate, and butyrate. These SCFAs are essential for gut health because they nourish gut cells, fight inflammation, and strengthen the gut barrier. Scientific evidence supports the role of SCFA-producing bacteria in the treatment of IBD [39,40]. These studies have shown that SCFA supplements can improve the symptoms of ulcerative colitis and Crohn’s disease, and that people with IBD who have higher levels of SCFA-producing bacteria are less likely to experience flares.

### Limitations

The weaknesses of this work include the heterogeneity of the studies reviewed, which makes the direct comparison of results and the generalization of conclusions difficult. In addition, most of the available studies are observational, which limits the ability to establish firm causal relationships between short-chain fatty acids and the immune regulation of IBD. It also faces the limitation of publication biases and variability in the study designs, methodologies, and populations studied. Finally, although the systematic review covers an extensive database, there may be relevant studies not included due to language or access restrictions, which could influence the completeness of the conclusions.

## 5. Conclusions

This study has shown that SCFAs, especially butyrate, play an important role in immune regulation in IBD. A systematic review of the literature has demonstrated that SCFAs may have a beneficial effect on reducing intestinal inflammation and modulating the immune response, suggesting their therapeutic potential in the treatment of IBD.

However, the heterogeneity of the reviewed studies poses certain limitations in generalizing the results. The variability in the study designs, populations studied, and methodologies used makes it difficult to make direct comparisons and draw definitive conclusions. It is essential that future studies address these limitations by using rigorous and standardized methodological designs and including diverse populations to validate and extend these findings.

Although the preliminary results are promising, further research is needed to confirm the therapeutic role of CCFAs in IBD and to establish clinically applicable treatment protocols. Future research should focus on conducting randomized clinical trials with representative samples and developing strategies for the clinical implementation of SCFAs to improve the outcomes in patients with IBD.

## Figures and Tables

**Table 1:** Summary of articles admitted for review.

Reference	Country	n	Objectives	Conclusions
Wilson B, 2021 [12]	UK	18 (UC)	To analyze the impact of prebiotic GOS on the inflammation, disease parameters, and microbiota of UC.	Prebiotics produce an increase in bifidobacteria in vitro and in vivo, which regulate pH by producing acetate and lactate, which favors butyrate production. There are low concentrations of bifidobacteria in intestinal inflammation. Both clinical scores and inflammation were not reduced with the use of these prebiotics. *Bifidobacterium* increased only in patients with lower disease activity.
Wei Y, 2016 [13]	China	20 (IBD)	To analyze the effects of pectin on FMT in UC patients.	Gut microbiota can ferment pectin into short-chain fatty acids. This maintains the composition and balance of the gut microbiota after FMT and preserves the diversity of the gut flora after FMT.
Facchin S, 2020 [14]	Italia	49 (IBD)	To analyze the effect of sodium butyrate microcapsules on the intestinal microbiota of patients with IBD and the regulation of intestinal bacterial formation, especially butyrogenic degenerogens and SCFA, by butyrate.	Oral supplementation with butyrate increases the production of intestinal SCFA-producing bacteria with anti-inflammatory activity, which will produce more endogenous butyrate for intestinal comfort. This treatment has been particularly beneficial in UC patients.
Pietrzak A, 2022 [15]	Polonia	79 (IBD)	To evaluate the effectiveness of sodium butyrate as an adjunct treatment in children and adolescents newly diagnosed with IBD.	Supplementation with sodium butyrate as an adjunctive therapy for 12 weeks did not show efficacy in children and adolescents newly diagnosed with IBD.
Chu ND, 2021 [16]	USA	8 (UC)	To analyze the dynamics of microbial colonization and persistence in UC patients treated with FMT, focusing on the transfer of butyrate production genes from a healthy donor to a patient.	There is an association between the loss of butyrate-producing microbes and IBD. There are difficulties in increasing the genetic capacity for butyrate production through fecal transplants.
Svolos V, 2018 [17]	UK	28 (UC)	To evaluate the effects on the gut microbiome, inflammation, and clinical response of an individualized food-based diet with a composition similar to EEN.	CD-TREAT mimics EEN changes in the microbiome and reduces intestinal inflammation. It is well tolerated and effective in patients with CD.
Luceri C, 2015 [18]	Italia	20 (IBD)	To study the effect of sodium butyrate enemas in preventing inflammation and mucosal atrophy in IBD patients after ileus/colostomy.	Butyrate enemas prevent colon/rectum atrophy and improve the recovery of tissue integrity.
Haskey N, 2023 [19]	Canada	38 (UC)	To investigate the differential effects of the Mediterranean dietary pattern (MDP) versus the Canadian dietary pattern (CHD) on the gut microbiome and inflammation in patients with inactive UC.	The MDP group had higher SCFA levels compared to the CDH group. MDP induces changes in the gut microbiome that are associated with clinical improvement in patients with inactive UC and may be used as an adjunctive therapy in patients with UC.
Hamilton AL, 2020 [20]	Australia	130 (IBD)	Identify the types of bacteria associated with the sustained remission of UC through fecal microbiota transplantation (FMT).	Butyrate and other SCFAs have been shown to induce an anti-inflammatory response in animal models and clinical trials. The efficacy of FMT in restoring butyrate-producing bacteria and ButCoA levels suggests an important role for butyrate in the long-term remission of UC and establishes butyrate or butyrate-producing bacteria as a potential treatment strategy.
Langhorst J, 2020 [21]	Germany	38 (UC)	To evaluate the effect of butyrate and other SCFAs in patients with UC with flares treated with mesalamine or a herbal preparation.	A decrease in SCFAs and butyrate has been observed in UC flare patients treated with mesalamine. Patients treated with the herbal preparation did not show this decrease in SCFAs.
Fritsch J, 2020 [22]	USA	17 (UC)	To study the effects of a low-fat, high-fiber diet (LFD) compared to a Standard American Diet (SAD).	Although the LFD diet had higher amounts of fiber than the iSAD, the only fatty acid that increased significantly was acetate. This has a beneficial effect on the intestinal barrier and inflammatory responses by binding to G protein-coupled receptors. LFD increases Bacteroides, which contain the main producers of acetate.
Ma HQ, 2018 [23]	China	15 (CD) 14 (UC)	To study alterations in the intestinal microbiota of Chinese patients with IBD.	Both *Butyricicoccus* and *Lachnobacterium* are SCFA-producing bacteria that act as an energy source for colonic epithelial cells. Changes in the gut microbiota, such as a decrease in these SCFA-producing bacteria, are associated with IBD patients.
Andoh A, 2012 [24]	Japan	161 (CD)	To study fecal microbiota profiles in patients with CD.	A decrease in the *Clostridium* class, including the genus *Fecalibacterium* (butyrate-producing bacteria), was found in the bacterial composition of CD patients.
O’Brien CL, 2013 [25]	Australia	47 (IBD)	To study the levels of *Fecalibacterium Prausnitzzi* and its relationship with CD.	Reduced levels of *Fecalibacterium prausnitzzi* have been observed in patients with CD, which is a cause and not a consequence of the disease. *Fecalibacterium prausnitzzi* and other fermenting organisms provide SCFAs, including butyrate, to the colonic epithelium, which uses it as an energy source. Butyrate plays an important role in reducing oxidative stress and as an anti-inflammatory agent. The more *Fecalibacterium prausnitzzi*, the better the gut ecosystem. The study found that inflammatory parameters were worse in patients with low *Fecalibacterium prausnitzzii*.
Fujimoto T, 2013 [26]	Japan	47 (IBD)	To study the relationship between dysbiosis in CD patients and the abundance of *Fecalibacterium prausnitzii* and *Bilophila wadsworthia* bacteria in Japanese patients.	The abundance of *Fecalibacterium prausnitzii* was significantly decreased in CD patients compared to healthy subjects. The decrease in *Fecalibacterium prausnitzii* results in a reduction of the anti-inflammatory activities regulated by these butyrate-producing bacteria.
Valcheva R, 2019 [27]	Canada	25 (UC)	To analyze whether insulin-type fructans produce benefits in UC and whether these are related to changes in bacterial composition.	B-fructan supplementation significantly increased SCFA production in the high-dose group but not in the low-dose group. B-fructans altered the composition of SCFAs. High-dose treatment with insulin-like fructans induces a modification of the gut microbiota, thereby improving UC and showing promise as a treatment for the disease.
Danilova NA, 2019 [28]	Russia	95 (IBD)	To study the composition of the gut microbiota in patients with IBD to identify key markers of dysbiosis in the disease.	The decrease in acetate CoA transferase, the decrease in the total number of SCFAs, as well as the particulate and major SCFA in IBD patients may indicate an inhibition of the functional activity and the amount of anaerobic microflora. These signs can be considered as typical for dysbiosis in IBD and can be used for the development of future treatments.
Ferrer-Picón E, 2020 [29]	Spain	137 (IBD)	To study the effects of butyrate on healthy and IBD-affected intestinal epithelium.	There is a reduction in butyrate-producing gut bacteria in patients with active IBD. The response to butyrate is not intrinsically altered in patients with IBD, but TNFα (a cytokine involved in the pathophysiology of IBD) produces a decrease in the epithelial response to this metabolite. This rejects the hypothesis of butyrate supplementation during active inflammation. The main effects of butyrate on the intestinal epithelium and the relevance of inflammation on the ability of epithelial cells to absorb, metabolize, and respond to this bacterial metabolite were determined.
Wang W, 2014 [30]	China	11 (CD) 20 (UC)	To compare the composition of fecal and mucosa-associated bacteria in patients with IBD and healthy controls.	Butyrate-producing bacteria of the *Clostridium* IV and XIVa groups were found to be decreased in IBD patients, particularly *F. prausnitzzi*. Previous reports have shown that *F. prausnitzii* produces butyrate and that its fermented product provides energy for colonic epithelial cells and is important for the immune system and epithelial barrier integrity. Butyrate-producing bacteria should be considered as probiotics for patients in the acute phase of IBD.
Lopez-Siles M, 2018 [31]	Spain	23 (UC) 37 (IBD)	To determine the variation of *A. muciniphila* and *F. prausnitzii* between healthy subjects and IBD patients.	In addition to butyrate production (which can reduce the inflammation of the intestinal mucosa and is the main energy source for colonocytes), other anti-inflammatory properties have been attributed to *F. prausnitzii*. IBD patients showed a decrease in this bacterium.
Lopez-Siles M, 2015 [32]	Spain	64 (IBD) 23 (UC)	To study whether patients with gastrointestinal diseases, particularly IBD, have differences in *F. prausnitzii* populations with respect to healthy subjects.	The population of *F. prausnitzii*, which is a part of the butyrate-producing bacteria group, is present in both healthy subjects and those with intestinal disease. However, there is a loss of abundance and altered distribution in patients with IBD.
Brotherton CS, 2016 [33]	USA	489 (UC) 1130 (CD)	To study whether dietary fiber intake is associated with exacerbations in IBD.	There are reasons to believe that fiber is beneficial for IBD patients because of the production of SCFAs, such as butyrate. Fiber intake is associated with reduced flares in CD but not in UC.
Kumari R, 2013 [34]	India	26 (UC)	To study fecal samples to determine the concentration of butyrate and the butyrate-producing bacteria in patients with UC.	With reduced butyrate levels, there is a decrease in members of the clostridial group that contribute to the etiology of UC. The decrease in butyrate-producing clostridia was associated with a decrease in SCFAs in UC patients.
Machiels K, 2014 [35]	Belgium	127 (UC)	To study the dysbiosis present in UC by quantifying bacterial metabolites.	SCFAs have been shown to be reduced in UC patients. The gut microbiota of UC patients shows a reduction in *R. hominis* and *F. prausnitzii*, known as butyrate-producing bacteria.
Takahashi K, 2016 [36]	Japan	78 (CD)	To study changes in the fecal microbiota in patients with CD.	In patients with CD, there is a decrease in butyrate-producing bacteria such as *Blautia faecis*, *Roseburia inulinivorans, Ruminococcus torques*, *Clostridium la-valense*, *Bacteroides uniformis*, and *Fecalibacterium prausnitzii*. This reduction in butyrate-producing bacteria characterizes the dysbiosis characteristic of CD patients.
Zhuang X, 2019 [37]	China	472 (IBD)	To characterize SCFAs in IBD patients and explore their potential role in initiating and developing IBD.	There are changes in CCFA in patients with IBD. There are inverse changes in CCFA in patients with active UC and those in remission.
Xu HM, 2022 [38]	China	4453 (IBD)	To study CCFA alterations in UC patients to investigate their role in disease pathogenesis.	Patients with UC have significantly lower concentrations of total SCFAs compared to healthy patients.
Cao Y, 2014 [39]	China	1180 (IBD)	To determine the relative risk of *F. prausnitzzi* decline in patients with and without IBD.	*F. prausnitzii* may protect against the development of IBD. Variation between studies and the possibility of bias preclude the certainty of this statement. Efforts are being made to use *F. prausnitzii* as an adjunct treatment for IBD by producing butyric acid.
Zhao H, 2021 [40]	China	1669 (IBD)	To study the relationship between intestinal *F. prausnitzii* and IBD.	There is a negative relationship between the amount of F. prausnitzzi and IBD activity. A cut-off level of *F. prausnitzzi* cannot be established to diagnose or initiate treatment for IBD.

SCFAs: short-chain fatty acids; EEN: Exclusive Enteral Nutrition; CD-TREAT: Crohn’s disease treatment with eating diet; CHD: Canadian dietary pattern; UC: ulcerative colitis; CD: Crohn’s disease; IBD: inflammatory bowel disease; FMT: fecal microbiota transplantation; GOS: galactolysaccharides; LFD: low-fat, high-fiber diet; MDP: Mediterranean diet pattern; SAD: standard enhanced American diet; pH: hydrogen potential.

## Data Availability

No new data were created or analyzed in this study.

## Supplementary Materials

The following supporting information can be downloaded at https://www.mdpi.com/article/10.3390/ijms252010879/s1.
