# Supplementary material for: Therapeutic and Immunologic Effects of Short-Chain Fatty Acids in Inflammatory Bowel Disease: A Systematic Review
Source: Int J Mol Sci. 2024 Oct 10;25(20):10879. doi: 10.3390/ijms252010879 (PMC11506931; doi:10.3390/ijms252010879)
Supplement: Supplementary file 1 [file ijms-25-10879-s001.zip › Supplementary Data S2.pdf]

Therapeutic and immunologic effects of short-chain fatty acids in inflammatory bowel disease: A systematic review.

Supplementary Material

Table S1. Search strategies

**Medline**	(("inflammatory bowel diseases"[MeSH Terms] OR ("inflammatory"[All Fields] AND "bowel"[All Fields] AND "diseases"[All Fields]) OR "inflammatory bowel diseases"[All Fields] OR ("inflammatory"[All Fields] AND "bowel"[All Fields] AND "disease"[All Fields]) OR "inflammatory bowel disease"[All Fields]) AND ("fatty acids, volatile"[MeSH Terms] OR ("fatty"[All Fields] AND "acids"[All Fields] AND "volatile"[All Fields]) OR "volatile fatty acids"[All Fields] OR ("short"[All Fields] AND "chain"[All Fields] AND "fatty"[All Fields] AND "acids"[All Fields]) OR "short chain fatty acids"[All Fields]) AND ("butyric acid"[MeSH Terms] OR ("butyric"[All Fields] AND "acid"[All Fields]) OR "butyric acid"[All Fields] OR "butyrates"[MeSH Terms] OR "butyrates"[All Fields]) AND ("butyrates"[MeSH Terms] OR "butyrates"[All Fields] OR "butyrate"[All Fields] OR "butyric"[All Fields])))
**Web of Science**	TS=(("inflammatory bowel diseases" OR ("inflammatory" AND "bowel" AND "diseases") OR "inflammatory bowel diseases" OR ("inflammatory" AND "bowel" AND "disease") OR "inflammatory bowel disease") AND ("fatty acids, volatile" OR ("fatty" AND "acids" AND "volatile") OR "volatile fatty acids" OR ("short" AND "chain" AND "fatty" AND "acids") OR "short chain fatty acids") AND ("butyric acid" OR ("butyric" AND "acid") OR "butyric acid" OR "butyrates" OR "butyrates") AND ("butyrates" OR "butyrates" OR "butyrate" OR "butyric"))

Table S2. Studies evaluated using the Joanna Briggs Institute critical appraisal checklist for quasi-experimental studies.

Study	Were the comparison groups found to be similar on the most important variable?	Were the participants analyzed as they were originally assigned to the groups?	Were comparisons made between individuals predisposed to receive or not receive the intervention?	Was the exposure or intervention measured in a standardized way?	Were the outcomes measured in a valid and reliable way?	Were appropriate statistical methods used in the analysis?	Were participants followed for a sufficient period of time?	Were outcomes measured reliably and consistently across raters?	Score out of 8 (100%)
Wilson B, 2021 [12]	N	N	N	Y	Y	Y	N	Y	4/8 (50%)
Wie Y, 2016 [13]	Y	Y	N	Y	Y	Y	Y	Y	7/8 (87.5%)
Facchin S, 2020 [14]	Y	Y	Y	Y	Y	Y	Y	Y	8/8 (100%)

Y = Yes. N = No. U = Unclear.

Table S3. Studies evaluated using the Joanna Briggs Institute critical appraisal checklist for randomized controlled trials.

Study	Was True Randomization Used for Assignment of Participants to Treatment Groups?	Was Allocation to Treatment Groups Concealed?	Were Treatment Groups Similar at the Baseline?	Were Participants Blind to Treatment Assignment?	Were Those Delivering Treatment Blind to Treatment Assignment?	Were Outcomes Assessors Blind to Treatment Assignment?	Were Treatment Groups Treated Identically Other Than the Intervention of Interest?	Was Follow up Complete and If Not, Were Differences between Groups in Terms of Their Follow up Adequately Described and Analyzed?	Were Participants Analyzed in the Groups to Which They Were Randomized?	Were Outcomes Measured in the Same Way for Treatment Groups?	Were Outcomes Measured in a Reliable Way?	Was Appropriate Statistical Analysis Used?	Was the Trial Design Appropriate, and Any Deviations from the Standard RCT Design (Individual Randomization, Parallel Groups) Accounted for in the Conduct and Analysis of the Trial?	Score out of 13 (100%)
Pietrzak A, 2022 [15]	Y	N	Y	N	Y	Y	Y	Y	Y	Y	N	Y	Y	10/13 76.9%
Luceri C, 2016 [18]	Y	Y	Y	Y	Y	Y	Y	Y	Y	Y	Y	Y	Y	13/13 100%
Haskey N, 2023 [19]	Y	N	Y	N	Y	Y	Y	Y	Y	Y	N	Y	Y	10/13 76.9%
Langhorst J, 2020 [21]	Y	N	Y	Y	Y	Y	Y	Y	Y	Y	N	Y	Y	11/13 84.6%
Fritsch J, 2021 [22]	Y	Y	Y	N	N	Y	Y	Y	Y	Y	N	Y	Y	10/13 76.9%
Valcheva R, 2019 [27]	Y	Y	Y	Y	N	Y	Y	Y	Y	Y	N	Y	Y	11/13 84.6%
Ferrer E, 2020 [29]	Y	N	Y	Y	Y	Y	Y	Y	Y	Y	N	Y	Y	11/13 84.6%

Y = Yes. N = No. U = Unclear.

Table S4. Studies appraised using the Joanna Briggs Institute critical appraisal checklist for cohort studies

	Were the two groups similar and recruited from the same population?	Were the exposures measured similarly to assign people to both exposed and unexposed groups?	Was the exposure measured in a valid and reliable way?	Were confounding factors identified?	Were strategies to deal with confounding factors stated?	Were the groups/participants free of the outcome at the start of the study or at the moment of exposure?	Were the outcomes measured in a valid and reliable way?	Was the follow up time reported and sufficient to be long enough for outcomes to occur?	Was follow up complete, and if not, were the reasons to loss to follow up described and explored?	Were strategies to address incomplete follow up utilized?	Was appropriate statistical analysis used?	Score out of 11 (100%)
Chu ND, 2021 [16]	N	Y	N	Y	N	N	Y	N	Y	Y	Y	6/11 54.5%
Svolos V, 2019 [17]	N	Y	Y	Y	Y	N	Y	N	Y	Y	Y	8/11 72.7%
Hamilton AL, 2020 [20]	Y	Y	N	Y	N	N	Y	N	Y	Y	Y	7/11 63.6%
Andoh A, 2012 [24]	N	Y	N	Y	N	N	Y	N	Y	Y	Y	6/11 54.5%
O'Brien CL, 2013 [25]	N	Y	Y	Y	N	N	Y	N	Y	Y	Y	7/11 63.6%
Fujimoto T, 2013 [26]	N	Y	N	Y	N	N	Y	N	Y	Y	Y	6/11 54.5%
Wang W, 2014 [30]	N	Y	N	Y	N	N	Y	N	Y	Y	Y	6/11 54.5%
Lopez M, 2018 [31]	N	Y	N	Y	N	N	Y	N	Y	Y	Y	6/11 54.5%
Lopez M, 2015 [32]	N	Y	Y	Y	N	N	Y	N	Y	Y	Y	7/11 63.6%
Brotherton CS, 2016 [33]	Y	Y	N	Y	N	N	Y	N	Y	Y	Y	7/11 63.6%
Kumari R, 2013 [34]	N	Y	N	Y	N	N	Y	N	Y	Y	Y	6/11 54.5%
Machiels K, 2014 [35]	N	Y	Y	Y	N	N	Y	N	Y	Y	Y	7/11 63.6%
Takahashi K, 2016 [36]	N	Y	N	Y	N	N	Y	N	Y	Y	Y	6/11 54.5%

Y: Yes; N: No; U: Unclear; NA: Not applicable

Table S5. Studies evaluated using the Joanna Briggs Institute critical appraisal checklist for analytic cross-sectional studies.

Study	Were the Criteria for Inclusion in the Sample Clearly Defined?	Were the Study Subjects and the Setting Described in Detail?	Was the Exposure Measured in a Valid and Reliable Way?	Were Objective, Standard Criteria Used for Measurement of the Condition?	Were Confounding Factors Identified?	Were Strategies to Deal with Confounding Factors Stated?	Were the Outcomes Measured in a Valid and Reliable Way?	Was Appropriate Statistical Analysis Used?	Score out of 8 (100%)
Ma HQ, 2018 [23]	Y	Y	Y	Y	N	N	Y	Y	6/8 (75%)
Danilova NA, 2019 [28]	Y	Y	Y	Y	N	N	Y	Y	6/8 (75%)

Y = Yes. N = No. U = Unclear.

Table S6. Studies evaluated using the Joanna Briggs Institute critical appraisal checklist for systematic reviews.

	Is the review question clearly and explicitly stated?	Were the inclusion criteria appropriate for the review question?	Was the search strategy appropriate?	Were the sources and resources used to search for studies adequate?	Were the criteria for appraising studies appropriate?	Was critical appraisal conducted by two or more reviewers independently?	Were there methods to minimize errors in data extraction?	Were the methods used to combine studies appropriate?	Was the likelihood of publication bias assessed?	Were recommendations for policy and/or practice supported by the reported data?	Were the specific directives for new research appropriate?	Score Out of 11 (100%)
Zhuang X, 2022 [37]	Y	Y	Y	Y	Y	N	Y	Y	N	Y	Y	9/11 81.8%
Xu XM, 2022 [38]	Y	N	Y	Y	N	N	Y	Y	N	Y	Y	7/11 63.6%
Cao Y, 2014 [39]	Y	Y	Y	N	N	N	Y	Y	N	Y	Y	7/11 63.6%
Zhao H, 2021 [40]	Y	Y	Y	Y	Y	Y	Y	Y	Y	Y	Y	11/11 100%

Y = Yes. N = No. U = Unclear.
